# Communication Self-Efficacy and Job Satisfaction among Nurses during the COVID-19 Pandemic

**DOI:** 10.1155/2024/8869949

**Published:** 2024-04-29

**Authors:** Leila Ghahremani, Zakieh Khoramaki, Mohammad Hossein Kaveh, Masoud Karimi, Mahin Nazari, Alejandro Orgambídez Ramos

**Affiliations:** ^1^Research Center for Health Sciences, Institute of Health, Department of Health Promotion, School of Health, Shiraz University of Medical Sciences, Shiraz, Iran; ^2^Student Research Committee, Shiraz University of Medical Sciences, Shiraz, Iran; ^3^Department of Health Promotion, School of Health, Shiraz University of Medical Sciences, Shiraz, Iran; ^4^Department of Social Psychology, University of Málaga, Málaga, Spain

## Abstract

**Introduction:**

The outbreak of COVID-19 has led to various challenges for healthcare workers, including nurses. Nurses play a critical role in the fight against this disease, and their communication of self-efficacy and job satisfaction has garnered significant attention. This study aimed to investigate the relationship between communication self-efficacy and job satisfaction of Iranian nurses during the COVID-19 pandemic.

**Methods:**

This study was conducted using a cross-sectional design. A total of 500 nurses working in hospitals in Iran were selected using a convenience sampling method. The communication self-efficacy scale and the job satisfaction scale were used to collect data.

**Results:**

The study found that nurses with higher communication self-efficacy exhibited better performance and job satisfaction in various work challenges (*r* = 0.56, *p* < 0.001). However, nurses holding a master's degree or higher reported the lowest average communication self-efficacy and job satisfaction scores during the COVID-19 epidemic. The study also explored the impact of shift work on job satisfaction among nurses and found that nurses working exclusively on the morning shift reported the highest average job satisfaction score.

**Conclusion:**

The findings of this study suggest that communication self-efficacy is an important factor in predicting job satisfaction among Iranian nurses during the COVID-19 pandemic. Therefore, it is recommended that healthcare organizations provide effective communication training sessions and mental health interventions to enhance nurses' communication self-efficacy and job satisfaction. This can ultimately lead to improved performance and better patient outcomes.

## 1. Background

With the outbreak of COVID-19 disease, various issues arose in the personal lives of nurses. Failure to fully follow preventive protocols in caring for COVID-19 patients could have turned the nurse into a carrier of the disease and endangered her family, and early onset of the disease led to feelings of fear and insecurity among nurses and their families [1, 2]. For a long time after the epidemic began, hospital staff neighbors always considered them carriers of the disease and avoided them, which led to more loneliness, fear, feelings of insecurity [3], and sometimes discouragement and regretted their choice of nursing profession and reduced job satisfaction [1].

### 1.1. Communication Self-Efficacy in Healthcare

Different functional situations in healthcare centers are often stressful and unpredictable [4], and a person's belief in his ability to master the correct and timely response shows the degree of individual self-efficacy. Communication self-efficacy is defined as a person's belief in his ability to communicate effectively in certain situations and the regulation of the thought processes, motivation, and physiological states required for effective communication in a particular situation. When assessing communication self-efficacy, people's communication skills are not measured but their confidence that they can successfully use any skill they have to communicate effectively in different communication environments [5, 6].

### 1.2. Nurse Job Satisfaction

Nurses have a great role to play in promoting global health, so it is necessary to plan and invest to improve their quality of life, which will definitely benefit society. By investing in the working conditions and quality of life of nurses, not only the job satisfaction of the nurse and the improvement of his performance will be provided but also the whole healthcare system and ultimately the whole society will benefit [7, 8].

### 1.3. Study Rationale

In light of the global COVID-19 pandemic, nurses have emerged as key and indispensable members in the fight against this disease [9]. Given their critical role in healthcare, the communication self-efficacy and job satisfaction of nurses have garnered significant attention [10]. Investigating the relationship between the communication self-efficacy and job satisfaction of nurses in the context of the COVID-19 pandemic can contribute to improving working conditions and enhancing nurses' performance in the fight against this disease [11]. Previous studies have demonstrated that nurses' self-efficacy and job satisfaction are closely linked to the quality of healthcare [12–14]. While context and certain social and physiological factors have the potential to influence the self-efficacy and job satisfaction of nurses, identifying these factors and implementing effective interventions can improve these two critical components. However, previous research has been limited by the use of conventional instruments to measure communication self-efficacy and job satisfaction, as well as a lack of consideration of critical situations like the COVID-19 pandemic. Furthermore, data on communication self-efficacy and job satisfaction among nurses during the COVID-19 pandemic are scarce. Therefore, the present study was conducted with the aim of investigating the relationship between communication self-efficacy and job satisfaction of nurses during the COVID-19 epidemic and the factors affecting these two components in this epidemic.

## 2. Materials and Methods

### 2.1. Design

All nurses deployed to Shiraz university hospitals to care for COVID-19 patients were eligible for this anonymous cross-sectional study.

### 2.2. Study Setting and Population

The research participations were the convenience population from the nursing staff of Shiraz public hospitals who had more than two years of work experience and were employed both before and after the COVID-19 era.

To calculate of sample size, we used the previous study [15], considering a 15% invalid questionnaire rate. Therefore, the final sample size was determined as 346.

### 2.3. Inclusion and Exclusion Criteria

Nursing staff working in hospitals, with more than two years of continuous work experience in the clinical wards of the hospital, no pregnancy, no long-term leave, and no specific diseases were among the inclusion criteria.

The exclusion criterion was the announcement of the nurse's withdrawal from continuing to participate in the study.

### 2.4. Instruments

#### 2.4.1. Participants' Demographic Profiles

Participants' demographic profiles included questions about age, sex, marital status, number of children, education level, years of work, care history of COVID-19 patients, etc.

#### 2.4.2. Communication Self-Efficacy during the COVID-19 Epidemic (CSE.C)

Questionnaire developed by researchers to measure CSE.C was used. The questionnaire was investigator developed based on an extensive literature review. Issues such as the nurse's ability to motivate the patient to describe his problems and concerns, active listening, appropriate nonverbal behaviors, empathy with the patient, checking the patient's awareness of the given information, and creating a plan based on shared decisions with the patient, etc. are stated in the existing questionnaire [16]. On the other hand, several articles have pointed out the importance of the nurse's ability to manage stress in critical situations, the ability to perform tasks with team coordination in critical situations, the ability to make a correct assessment in unexpected events, etc. In the existing communication self-efficacy questionnaire, these dimensions are not mentioned [17–19]. For this reason, we decided to design special questions to measure the communication self-efficacy of nurses during the COVID-19 era. The CSE.C questionnaire was evaluated with 19 questions with a score of 1 from “not at all sure” with a score of 10 to “absolutely sure” with a score of 1, which assesses a person's perception of competence and ability to deal effectively with stressful situations such as COVID-19 era. The total score of this questionnaire is 19–190, and the higher the score, the higher the self-efficacy.

#### 2.4.3. Job Satisfaction during the COVID-19 Epidemic (JS.C)

Questionnaire developed by researchers to measure JS.C was used. The questionnaire was investigator developed based on an extensive literature review. It was necessary to select a questionnaire that would measure the different dimensions of job satisfaction among nurses during the COVID-19 era with the fewest questions. Therefore, based on the review of the literature, the existing questionnaires are either designed only for normal working conditions or for other dimensions of job satisfaction in crisis conditions, such as benefits commensurate with the volume and difficulty of the work, managers' understanding of the efforts and tolerance of the hardships of nursing work, and timely encouragement and appreciation, as well as satisfaction with their ability to provide quality nursing care to patients with COVID-19, have mentioned a little. Therefore, the research team decided to design a questionnaire to measure the job satisfaction of nurses during the COVID-19 era. JS.C questionnaire was evaluated with 8 questions and six-point scoring from a score of one (strongly disagree) to six (strongly agree).

#### 2.4.4. Reliability and Validity

Questionnaire's face and content validity were evaluated by 9 faculty members in the fields of nursing and health education and promotion. Then, based on their opinions, necessary changes were made in CSE.C questionnaire (CVR 0.78 and CVI 0.79) and JS.C questionnaire (CVR 0.76 and CVI 0.82). The reliability of the tool was evaluated through the retest method, which was completed on two occasions with an interval of two weeks by 21 nurses working in other hospitals. The reliability coefficient of the retest results after two weeks was 0.805 and 0.667, respectively, and Cronbach's alpha coefficient was 0.93 and 0.72 (*P* < 0.001).

### 2.5. Procedure

In order to collect information, while providing a complete description of the project, the questionnaires were provided to the target group online. At the beginning of the questionnaire, the consent form and the purpose of the project were mentioned that the individual's signature and completion of the information meant the individual's consent to participate in the research.

### 2.6. Statistical Analysis

The study data were analyzed using the SPSS statistical software, version 26. Data analysis and description of variables were performed using descriptive statistics including mean indices, standard deviation, and analytical statistics including independent *T*-test, ANOVA, and Pearson's correlation tests. *P* < 0.05 was considered as statistically significant.

### 2.7. Ethical Considerations

Ethical approval was obtained from Shiraz University of Medical Sciences and Shiraz Ministry of Education. Moreover, considering that the data collection was online, the participants were asked to read the full description of the study that we posted at the beginning of entering the site, and if they want to participate, select the option “consent to participate in the study.”

Ethics Committee code: (IR.SUMS.SCHEANUT.REC.1400-068).

## 3. Results

A total of 500 nurses participated in the study and all completed questionnaires were analyzed. Most participants were female (79.8%), married (61.2%), and had a bachelor's degree (86.8%). Their mean age was 33.74 ± 6.8 years, work experience was 9.49 ± 6.44 years, and 44.6% of them mentioned the history of caring for Covid patients in the hospital.

The results of univariate analysis showed that there is no statistically significant relationship between most of the demographic and work characteristics of nurses (sex, age, education level, number of children, working years, work shift, and care history of COVID-19 patient) with their communication self-efficacy and job satisfaction. The only statistically significant relationship obtained in this study was between the marital status and communication self-efficacy of nurses. The highest mean score of the communication self-efficacy was obtained for the group of widowed/divorced nurses (164.58 ± 22.84). Despite the lack of significant results for average communication self-efficacy and job satisfaction scores, it should be noted that nurses holding a master's degree or higher reported the lowest average communication self-efficacy and job satisfaction scores during the epidemic, respectively (132.47 ± 32.88 and 27.31 ± 7.21).

The results derived from the job satisfaction scores obtained during the COVID-19 pandemic indicate that nurses working exclusively in the morning shift reported the highest average job satisfaction score (29.21 ± 5.22) (Table 1).

Mean and standard deviation of nurses' CSE.C questionnaire total scores were 142.75 ± 30.41 and JS.C scores of 28.11 ± 6.48 were obtained (Table 2).

Table 3 shows the maximum and minimum average score of the participants' answers to the CSE.C and JS.C questions.

The Pearson's correlation test showed positive and significant relationships between CSE.C and JS.C (*r* = 0.254, *P*=0.000). Nurses who had higher communication self-efficacy in caring for the COVID-19 patient also reported almost higher job satisfaction during the COVID-19 epidemic. Also, a statistically significant correlation was found between age, work shift, and CSE.C (Table 4).

## 4. Discussion

This study investigated the relationship between communication self-efficacy and job satisfaction during the COVID-19 epidemic among hospital nurses who worked in Shiraz university hospitals.

### 4.1. Key Findings

#### 4.1.1. Comparison between CSE.C and JS.C Scores among Participants' Demographic Characteristics

The results showed that CSE.C has a significant statistically relationship with the marital status. In Motahari et al.'s study, married students had higher clinical self-efficacy than unmarried students, which is not consistent with the results of the present study [20]. The findings of Asadpour and Sadat Hosseini's study showed that normal women and men have higher self-efficacy compared to divorced people [21], which is not consistent with the results of the present study. To date, research has not consistently shown that the self-efficacy of widowed and divorced nurses, or other widowed and divorced individuals, is higher than single and married people. However, studies that have implemented educational interventions for these groups have demonstrated positive outcomes in terms of mental health and empowerment [21–23]. It is possible that the high levels of communication self-efficacy observed in nurses during the COVID-19 pandemic can be attributed to their participation in effective communication training sessions and mental health interventions. Further research is required to confirm this hypothesis.

Nurses holding a master's degree or higher reported the lowest average communication self-efficacy and job satisfaction scores during the COVID-19 epidemic. However, there are no specific studies that provide positive or negative results regarding this finding. Nevertheless, there are several studies that have investigated factors affecting job satisfaction and burnout among nurses. For example, one study found that job satisfaction was positively related to professional commitment among nursing students during the COVID-19 lockdown [24]. Another study found that job satisfaction was negatively affected by toxic leadership practices among nurses [25]. In addition, a study found that burnout among nurses was related to their level of self-efficacy and job satisfaction [26]. It is important to consider factors such as job satisfaction, self-efficacy, and leadership practices when addressing the well-being of nurses during the COVID-19 pandemic [24–26].

The results indicate that nurses working exclusively in the morning shift reported the highest average JS.C score. Several studies have investigated the effect of shift work on the job satisfaction of nurses. One study conducted in Pakistan found that nurses working rotating shift duties reported lower job satisfaction than those working day shifts [27]. Another study conducted in Italy found that nurses working rotating night shifts reported the lowest mean score in job satisfaction compared to those working day shifts [28]. The available evidence suggests that shift work, particularly rotating night shifts, can have a negative impact on the job satisfaction of nurses. More research is needed to fully understand the relationship between shift work and job satisfaction among nurses during the COVID-19 pandemic.

The results indicated a statistically significant correlation between age and CSE.C. Lim et al. in their study indicated that significantly nurses aged 50 years or more have high self-efficacy [10]. Also, Mehralian et al. in their study on 312 nurses working in a COVID-19 hospital in the south of Iran; they also reached a similar conclusion [29]. Therefore, it can be concluded that age is one of the important factors in nurses' self-efficacy against COVID-19. Older nurses show greater self-efficacy against this disease. This issue can be considered as one of the important components in the planning and management of human resources in the field of nursing.

Finally, the relationship between sociodemographic characteristics of nurses and their self-efficacy and job satisfaction appears to be complex and multifactorial. It is important for nursing managers to consider these factors and construct a monitoring system to reduce negative outcomes and improve job satisfaction and self-efficacy among nurses.

#### 4.1.2. Nurses' Communication Self-Efficacy during the COVID-19 Epidemic

The study findings revealed that the highest average response of the participants to the CSE.C questions was obtained in relation to the question “in any situation, especially critical situations such as caring for a COVID-19 patient, how confident are you in coordinating your duties with the team?” Based on the search results, there is limited information on the confidence of nurses in their ability to perform teamwork in critical situations such as taking care of a COVID-19 patient. However, there are studies that explore the influence of management modes on the teamwork ability of nurses [30]. In addition, newly graduated registered nurses working in acute care hospital settings play a critical role in providing safe nursing care, and teamwork is essential in reducing preventable errors and improving safety outcomes [31]. While there is limited information on the confidence of nurses in their ability to perform teamwork in critical situations such as taking care of a COVID-19 patient, teamwork is essential in providing safe nursing care and reducing preventable errors.

The lowest average response of the participants to the communication CSE.C was obtained in relation to the question “how confident are you in your ability to successfully prepare and conduct a preprepared conversation plan with a COVID-19 patient?” There is limited evidence available on nurses' confidence in developing a conversation plan for critical situations in general. However, some studies have explored the impact of the COVID-19 pandemic on communication in clinical settings and the use of communication strategies in online teaching and learning during the pandemic. Communication was slightly affected during the pandemic, especially nonverbal communication, with verbal communication maintained and, in some cases, strengthened [32]. In terms of end-of-life care planning, the use of the Supportive and Palliative Care Indicators Tool (SPICT) for hospital admissions and the application of education in topics related to end-of-life care resulted in a significant improvement in renal nurses' perception of confidence in their ability to recognize end of life [33]. While there is limited information available on nurses' confidence in developing a conversation plan for critical situations in general, studies have explored the impact of the COVID-19 pandemic on communication in clinical settings and the use of communication strategies in online teaching and learning during the pandemic. In addition, studies have explored the use of tools such as SPICT to improve nurses' confidence in recognizing end-of-life care needs.

#### 4.1.3. Nurses' Job Satisfaction during the COVID-19 Epidemic

The highest average response of the participants to the JS.C questions was obtained in relation to the question “I am satisfied with my ability to influence quality nursing care of patients?” A study conducted in Quebec, Canada, found that nurses caring for COVID-19 patients reported high chronic fatigue, poor quality of care, lower work satisfaction, and higher intention to leave their organization [34]. However, another study found that nurse-led multidisciplinary team care, training, and development, appropriate skill mix, quality, and outcome of care can lead to improved nurse-patient relationships and increased satisfaction with the quality of care provided [35]. Effective communication and support for nurses are crucial in providing quality care to COVID-19 patients. It is also important to address concerns about nurse-led care and provide evidence regarding patient safety, clinical outcomes, cost, and patient satisfaction to reflect on the ability of nurses to provide high-quality care within the primary care setting [36].

The lowest average response of the nurses to the JS.C questions was obtained in relation to the question “the salary and benefits received are proportional to the volume and difficulty of my work during the COVID-19 pandemic.” One study found that financial factors, such as monthly salary or bonuses, had no or little impact on work motivation during the pandemic [37]. It appears that the impact of COVID-19 on nurse salaries and benefits is complex and multifaceted, with various factors affecting compensation and job satisfaction. This emphasizes the importance of nursing organizations playing an active role in advocating for the profession and ensuring that nurses' voices are appreciated in the healthcare system.

#### 4.1.4. Correlation Relationship between CSE.C and JS.C

The correlation results showed a positive and significant relationship between CSE.C and JS.C in the nurses under study. The results of studies conducted on employees of different jobs showed that self-efficacy is one of the factors affecting job satisfaction [12, 38–40]. Similar results were obtained in the studies of Klassen et al. on 1430 Canadian teachers [41], and Darvish et al. on 100 Tabrizi-Iranian nurses [42]. People who believe in their abilities act well in the face of challenging situations, which will make them feel productive and increase their job satisfaction. Nurse self-efficacy is a key factor in how to care the patient and his job satisfaction. The nurse's performance during stressful situations such as the COVID-19 epidemic may overshadow the individual's self-efficacy. A reasonable understanding of the consequences of COVID-19 in nurses and anticipation of possible measures to deal with these consequences is important in their effective management [3].

### 4.2. Practical Implications

The results of this current study can furnish substantial evidence for nursing managers, decision-makers, and policymakers in the nursing field, augmenting their knowledge of the factors that influence nurses' communication self-efficacy and job satisfaction. Moreover, this study can serve as a commendable initial step towards designing and implementing programs aimed at enhancing nurses' communication self-efficacy and job satisfaction. In addition, as this study was conducted during the COVID-19 pandemic, its outcomes can provide a reliable benchmark for future research, both during and after critical situations and for studies conducted under normal and non-critical circumstances.

### 4.3. Limitations and Future Research

To increase the generalizability of the results, future research should be improved with a more balanced mix of male and female nurses. Also, the tool used in this study was a self-report questionnaire that could potentially impair data quality due to social desirability. Despite the limitations, in the present study, two questionnaires of self-efficacy and job satisfaction were developed during COVID-19 and, according to their validity and reliability, can be used in similar epidemics.

## 5. Conclusions

The relationship between sociodemographic factors and nurses' self-efficacy and job satisfaction is complex and multifactorial. Therefore, nursing managers need to take these factors into account and establish a monitoring system to improve job satisfaction and self-efficacy among nurses. In addition, factors such as widowhood, divorce, shift work, and communication strategies can affect nurses' job satisfaction and self-efficacy during the COVID-19 pandemic. Furthermore, nursing organizations need to advocate for the profession and ensure that nurses' voices are heard in the healthcare system. Finally, the age of nurses appears to be an important factor in their self-efficacy against COVID-19, with older nurses showing greater self-efficacy. This finding can be considered in the planning and management of human resources in the nursing field. Overall, an understanding of the complex factors affecting nurses' job satisfaction and self-efficacy is crucial for effective management and care during the COVID-19 pandemic.

## Figures and Tables

**Table 1:** Associations of sociodemographic variables with nurse's CSE.C and JS.C (*n* = 500).

Characteristics	*n* (%)	CSE.C score	JS.C score
		Mean ± SD	Mean ± SD
*Sex*			
Female	399 (79.8)	143.46 ± 29.81	28.08 ± 6.34
Male	101 (20.2)	139.92 ± 32.67	28.21 ± 7.02
*P* value^a^		0.771	0.154

*Age group*			
22–32 years	218 (43.6)	139.33 ± 29.31	27.73 ± 6.43
33–43 years	243 (48.6)	145.24 ± 31.98	28.37 ± 6.63
44–55 years	39 (7.8)	146.33 ± 24.54	28.56 ± 5.84
*P* value^b^		0.084	0.511

*Education level*			
Associate degree	34 (6.8)	147.12 ± 28.97	28.65 ± 7.02
Bachelor	434 (86.8)	143.16 ± 30.25	28.12 ± 6.39
≥Master of Science	32 (6.4)	132.47 ± 32.88	27.31 ± 7.21
*P* value^b^		0.109	0.699

*Marital status*			
Married	306 (61.2)	143.43 ± 29.48	28.18 ± 6.45
Single	182 (36.4)	140.15 ± 31.84	27.96 ± 6.68
Widowed/divorced	12 (2.4)	164.58 ± 22.84	28.50 ± 3.72
*P* value^b^		0.021^*∗*^	0.917

*Number of children*			
0	265 (53)	142.83 ± 30.23	27.83 ± 6.45
1	121 (24.2)	145.38 ± 31.92	28.45 ± 6.86
2	101 (20.2)	139.88 ± 28.14	28.50 ± 6.16
≥3	13 (2.6)	138.85 ± 37.59	27.54 ± 6.28
*P* value^b^		0.570	0.739

*Working years*			
2–10 years	270 (54)	140.96 ± 29.85	27.87 ± 6.29
11–20 years	206 (41.2)	144.27 ± 31.99	28.45 ± 6.85
21–30 years	24 (4.8)	149.83 ± 20.17	27.83 ± 5.23
*P* value^b^		0.252	0.621

*Work shift*			
Morning	33 (6.6)	144.52 ± 28.21	29.21 ± 5.22
Morning/evening	38 (7.6)	152.18 ± 26.85	29.03 ± 7.34
Rotation	429 (85.8)	141.78 ± 30.78	27.94 ± 6.48
*P* value^b^		0.122	0.368

*Care history of COVID-19 patient*			
Yes	223 (44.6)	143.23 ± 31.09	27.93 ± 6.39
No	277 (55.4)	142.36 ± 39.90	28.25 ± 6.56
*P* value^a^		0.731	0.810

^*∗*^ *p* < 0.05, ^a^independent *T*-test, ^b^ANOVA.

**Table 2:** Mean and standard deviation of CSE.C and JS.C

Variables	Mean	SD	Min. score	Max. score
CSE.C	142.75	30.41	19	190
JS.C	28.11	6.48	8	48

**Table 3:** The maximum and minimum average response of the participants to the CSE.C and JS.C questions.

Instrument	Questions	Scores	Mean ± SD
CSE.C	(i) In any situation, especially critical situations such as caring for a COVID-19 patient, how confident are you in coordinating your duties with the team	Maximum	7.96 ± 1.93
	(ii) How sure are you that you are successful and capable in preparing a preprepared conversation plan with the patient of COVID-19?	Minimum	6.33 ± 2.42

JS.C	(i) I am satisfied with my ability to influence quality nursing care of patients	Maximum	4.85 ± 1.01
	(ii) The salary and benefits received are proportional to the volume and difficulty of my work	Minimum	1.80 ± 1.33

**Table 4:** Correlation coefficients between research variables (*n* = 500).

Variables	CSE.C	
	*r*	*P*
(1) Age	0.094	0.036^*∗*^
(2) Work shift	0.090	0.045^*∗*^
(3) JS.C	0.254	0.000^*∗∗*^

^*∗∗*^Correlation is significant at the 0.01 level (2 tailed). ^*∗*^Correlation is significant at the 0.05 level (2 tailed).

## Data Availability

The data used to support the findings of this study may be released upon application to the Department of Health Education and Health Promotion, who can be contacted at healthpromotion.sums@gmail.com.
